# London Hospital Nurses

**Published:** 1918-08-03

**Authors:** Helena McKinley

**Affiliations:** London, E.


### THE LONDON HOSPITAL NURSES.

### London Hospital Nurses.

*To the Editor of* THE HOSPITAL.

SIR,—I, with many others, am very glad to see your letters of July 13 and 16 in this week's HOSPITAL with reference to the London Hospital training. It has been a common cause of comment among nurses that in all Lord Knutsford's camouflages you have practically always supported him, and, as you are Editor-in-Chief of THE HOSPITAL and other nursing publications, as well as a firm supporter of our College, it has been most difficult to understand your attitude. Naturally your association with the "London" (which I did not know of till now) will explain your interest somewhat; but it does not explain why, as one of the governors, you were not more associated with the training and well-being of the "London" nurses. As a matron of some years' standing, I have steadfastly refused to engage a "London" nurse, and I know of many matrons who do likewise. I always tell them personally I consider them untrained. Practically always their work is superficial and showy, and if fault is found with their methods they quote: "We always do this or that at the 'London.'" Nor are they usually loyal to the hospital or matron they work for. *But* is it true that they get *even two* years' work in the wards? I know many "Londoners" who have complained of lack of work and teaching, and who have been sent out private nursing under twelve months. There are also times when a woman who has nice manners and a pleasing presence is made sister of a ward long before two years' work has been done. . . . There are many other points which practically the whole nursing world have against the "London" and its methods. It is usually considered "outside the pale" by all those who know anything of the hospital nursing world.

I have had no opportunity of discussing your letters except with the matron here; but I assure you that you will earn the thanks not only of nurses outside the "London," but of the "Londoners" themselves, if you will take up their cause and secure for them what they are too young and inexperienced to know for themselves when they enter for their (?) training. "Exploitation" is the right word.—Yours faithfully, HELENA MCKINLEY.

London, E., July 27, 1918.

